# Supplementary material for: Soil bacterial assemblage responses to wildfire in low elevation southern California habitats
Source: PLoS One. 2022 Apr 8;17(4):e0266256. doi: 10.1371/journal.pone.0266256 (PMC8992989; doi:10.1371/journal.pone.0266256)
Supplement: Attachment — Submitted filename: Coments.docx [file pone.0266256.s002.docx]

Dear Author

The MS is of high quality and technically sound. However various questions arise after following the study parameters.

1. The richness and diversity of bacteria are dependent on both biotic and abiotic factors. In the present study, only fire has been taken to see the effect on bacteria community. How this study can neglect the effect of other parameters while studying in the open ecosystem.
2. As per your hypothesis, wildfire should negatively affect the microbial diversity but your findings suggest that the effect was positive or negligible. What are the chances that these effects are not because of fire but may be due to microbial interaction with other abiotic factors like edaphic factors?
3. The study does not have control, therefore the results do not seems reliable or you can say it may have other influences too.
4. It has been mentioned that diversity was higher in 2019 (post-fire) than 2017 (Pre-fire) but the diversity did not differ between 2016 and 2019. It means in 2017, diversity had fluctuated. Any reason?
5. Fire usually affects various soil properties which ultimately affects the bacterial community. In this study, soil parameters have not been studied at concerned site.
6. Do the studied sites have frequent wildfire history? The bacterial populations of the concerned sites may have resistant bacteria whose diversity has been modulated by wildfire in the past history before your study.
7. With the sequencing data, what could be elaborated is not reflected in result and discussion portion.
8. MS is not written in crispy fashion. Too large introduction, material methods and discussion.
9. Sometimes sentences are too large to understand like line number 81 to 85.
10. Rectify the line number 169, 30-36 30s cycles at 94^0^ C?
11. The application of the study has not been reflected in abstract and discussion portion. Please look into that.
12. Check the references as per journal guidelines.
