# Supplementary material for: Soil bacterial assemblage responses to wildfire in low elevation southern California habitats
Source: PLoS One. 2022 Apr 8;17(4):e0266256. doi: 10.1371/journal.pone.0266256 (PMC8992989; doi:10.1371/journal.pone.0266256)
Supplement: Attachment — Submitted filename: Response to Reviwers - Cox et al..docx [file pone.0266256.s003.docx]

**Response to Reviewers**

*We appreciate the reviewers’ efforts. Below each comment, you will find our response in red and italics. Of course, if we misinterpreted anything, we would be happy to make further revisions. In a few instances we were collectively confused about what the reviewer was requesting but attempted to do our best to clarify any confusion. Overall, it seemed like reviewer 2 was a little confused about the methodological approach, so we focused significant editorial attention on that section.*

1. Reviewer #1: Manuscript recommended for publication
   1. Manuscript reserch results indicate that while direct effects of fire in low elevation southern California systems are minimal, indirect effects, changes in plant community structure resulting from the disturbance, could have significant long-lasting legacy impacts on soil bacterial assemblage structure are remarkable

*Thank you. We mention work on plant soil-feedbacks in this manuscript and legacy effects associated with modifications in plant assemblages. What we find interesting is that these last even after a major disturbance such as fire, something we did not expect when we first designed this experiment.*

2.Richness and diversity increased following the fire highlighting that fires do not lead to declines in bacterial ASV richness and diversity. While increased richness and diversity following a fire suggests that fires may facilitate bacterial recruitment, these changes only minimally impacted bacterial assemblage structure, with no differences being found between 2017 (pre-fire) and 2019 (post-fire) samples in three of the six habitats sampled. These findings are remarkable.

*We were initially surprised by these results. However, upon reflection, we considered that ground dwelling invertebrates also survive fire events. If they can survive fire events, it suggests that bacteria below the soil surface will also likely survive these disturbance events as conditions are likely not modified enough to limit life. Also, it is likely that bacteria that have evolved in harsh hot-dry conditions may be particularly well adapted at surviving fire. We highlight these ideas in the manuscript as it may seem counterintuitive. However, we also hope this spurs further research that can form a predicative framework for understanding the effects of fires in southern California.*

3.Please explain in about DNa isolation method incase you did any modification in refered procedure?

*We clarified that we used the exact same method as that of Caspi et al. 2018. Also, we focused efforts on editing this section to alleviate any ambiguity that may have existed.*

4.Note that Bioproject PRJNA398660 contains samples from other sites, only the bacterial samples from the Santa Monica Mountains site were used in this study. A list of these specific Biosamples is available in the maps directory in the paper’s GitHub repository: <https://github.com/aroc110/Cox-et-al-2021.Pl> improve this centence

*We rewrote this paragraph to make it clearer and also added a supplementary file with the accession numbers of all Biosamples used.*

5.How can fire affect bacterial and biological diversity pl clarify in discussion

*We apologize, but we are unclear how to address this comment. Our manuscript’s focus was on how fire and modifications influence bacterial diversity.*
6.Pl explain about future prospective also

*We apologize, but we are unclear what specifically you are asking us to do. Our manuscript addresses how modifications in bacterial assemblages influence plant-soil feedbacks and ecosystem function and we present the idea that bacteria in hot-dry systems and in areas with regular fire may be preconditioned to survive a fire. We are not sure what other perspectives you would like us to provide.*

7.Pl elaborate methodology

*We modified some of the methods section to make it more clear. In particular, we tried to clarify the DNA extractions and sequencing section. We also want to highlight that additional information and details, which we do not find critical to the manuscript, but maybe of interest to individuals aiming to use or replicate our results, are available in the GitHub repository and in the newly added S2 Table.*

2. Reviewer #2: Dear Author
   The MS is of high quality and technically sound. However various questions arise after following the study parameters.

*Thank you. I think we can address your concerns.*

1. The richness and diversity of bacteria are dependent on both biotic and abiotic factors. In the present study, only fire has been taken to see the effect on bacteria community. How this study can neglect the effect of other parameters while studying in the open ecosystem.

*We agree that bacterial richness, diversity, and assemblage structure are all influenced by many different factors. While we did not directly address each of these factors, they are incorporated into the experimental design which aimed to examine the direct impacts of fire on soil bacterial assemblages. In this study, we had 3 different sites which incorporated variation in local abiotic conditions and differences in soil properties. Pre-fire differences in soil abiotic and biotic properties across sites and habitats can be obtained from Caspi et al. 2019. In addition, at one site, we had two years of pre-fire data which allowed us to examine how interannual fluctuations in bacterial assemblages between years without fire and compare how bacterial assemblages differed immediately following a fire. We agree that had we found large shifts in richness, diversity, and assemblage structure following a fire that further exploration examining how these properties were influenced would be critical to identifying mechanisms driving differences in bacterial assemblages. However, we found that fire had minimal effects of bacterial diversity and assemblage structure at all three sites, and that fluctuations after the fire were consistent with interannual variation observed between years without a fire disturbance. Combined, our results highlight that despite differences across sites and habitats with varying soil conditions, there were consistent patterns associated with fire effects.*

3. As per your hypothesis, wildfire should negatively affect the microbial diversity but your findings suggest that the effect was positive or negligible. What are the chances that these effects are not because of fire but may be due to microbial interaction with other abiotic factors like edaphic factors?

*Again, there is significant diversity in edaphic (soil) factors across sites and habitat types. Despite this, we found a relatively consistent pattern that richness increased following the fire, a pattern that was contrary to our initial hypotheses. The fact that the patterns were consistent across sites and habitats with varying soil and abiotic properties and did not support our hypothesis derived from synthesis of the literature is interesting as it suggests that research from other areas may not predict what is happening at our study site. As for identifying the mechanism for this increase, we did highlight that Perez-Valera et al. also found that phylogenetic diversity increased following a fire suggesting potentially increased colonization rates. However, specifically saying anything about the complex interactions among bacteria and soil and abiotic factors would be conjecture.*

3. The study does not have control, therefore the results do not seems reliable or you can say it may have other influences too.

*We allocate an entire paragraph (2^nd^ to last paragraph in the Discussion) to describing the logistical difficulties associated with studying the impacts of wildfire. We do not agree that a control site would provide important insights, especially as we did not have pre-fire data for other sites. Also, we have found that sites close together (< 1 km) differ significantly in their microbial assemblages (see Caspi et al. 2018). Consequently, we argue that obtaining data from sites outside the fire scar would not provide any useful data on the impacts of fire, but instead explore how bacterial assemblages are structured geographically, not a question addressed here. Another thing to consider, is that fire studies with controls are normally associated with controlled burns that happen at cooler times of the year and have fires that are less intense than wildfires. Because we have pre- and post-fire samples at the same exact sampling sites (± a few meters due to GPS accuracy) within a wildfire, we believe our results are more predictive of the effects of fire than most manipulative fire studies. Our study is observational and not manipulative, which can bother some people, but we argue that our study sites, composed of natural areas that burned in a wildfire, provide better insights into effects of wildfires than most manipulative experiments would.*

4. It has been mentioned that diversity was higher in 2019 (post-fire) than 2017 (Pre-fire) but the diversity did not differ between 2016 and 2019. It means in 2017, diversity had fluctuated. Any reason?

*It is an interesting question. Unfortunately, we have no idea what factors were responsible for a decrease in bacterial diversity in 2017 at our Santa Monica Mountain site. However, the most important aspect of this finding is that it provides insights into how bacterial assemblages are changing across years without a fire disturbance. Understanding interannual variation allows us to better interpret post-fire findings. A wild hypotheses would be that drought (2016) and fire (2019) reduce abundances of dominate bacteria taxa allowing increased recruitment of other bacteria taxa. Increased ASV richness may then increase diversity. However, this hypothesis is not supported as ASV richness increased only in the non-native grassland and not the sage scrub habitat, leaving us to wonder why diversity was also reduced in the sage scrub habitat in 2017.*

5. Fire usually affects various soil properties which ultimately affects the bacterial community. In this study, soil parameters have not been studied at concerned site.

*See response to comment 1 by this reviewer, where they expressed similar concerns. Briefly, we found minimal differences in bacterial diversity and assemblage structure before and after a fire. Consequently, even if soil properties did change, they had minimal impacts on bacterial assemblages. Also, hypotheses derived from synthesis of the literature were not supported highlighting that patterns observed elsewhere can not be applied to our study system and that fire may be having different effects that may be presumed.*

6. Do the studied sites have frequent wildfire history? The bacterial populations of the concerned sites may have resistant bacteria whose diversity has been modulated by wildfire in the past history before your study.

*Again, this is an interesting question, but anything we add here would be conjecture. In the discussion we discuss a variety of factors that may precondition bacterial assemblages in our study sites to survive a fire. However, to test if fire history is playing a role, we would need increased number of sites with varied fire frequency histories. I think this is something that should be addressed, and we highlight that in the discussion.*

7. With the sequencing data, what could be elaborated is not reflected in result and discussion portion.

*We do not understand how to address this. Everything we present is associated with the sequencing data.*

8. MS is not written in crispy fashion. Too large introduction, material methods and discussion.

*As recommended, we have gone through the manuscript and modified the writing as best we could to make it more concise. We argue that the size of this manuscript and the relevant sections, are consistent with many other manuscripts and necessary to synthesize the literature and support our hypotheses and explain all the nuisance of exploring these complex questions.*

9. Sometimes sentences are too large to understand like line number 81 to 85.

*We agree. We have made modifications throughout the manuscript and modified this sentence as requested. In other instances, we have shortened or divided compound sentences throughout the manuscript.*

10. Rectify the line number 169, 30-36 30s cycles at 940 C?

*We agree that this was a needed modification. Thank you.*

11. The application of the study has not been reflected in abstract and discussion portion. Please look into that.

*We are unsure what we failed to do. To rectify any issues, we paid special attention to the final paragraph of our discussion and made some changes to make the application more apparent. We did not modify the abstract as we believe it highlights the most important aspects associated with the interpretation of our results.*

12. Check the references as per journal guidelines.

*We went through the references and made sure that they follow the journal guidelines.*
